# Clinical and genetic characteristics of carriers of the *TP53* c.541C > T, p.Arg181Cys pathogenic variant causing hereditary cancer in patients of Arab-Muslim descent

**DOI:** 10.1007/s10689-024-00391-2

**Published:** 2024-05-14

**Authors:** Johnathan Arnon, Aviad Zick, Myriam Maoz, Nada Salaymeh, Ahinoam Gugenheim, MazalTov Marouani, Eden Mor, Tamar Hamburger, Nagam Saadi, Anna Elia, Gael Ganz, Duha Fahham, Amichay Meirovitz, Luna Kadouri, Vardiella Meiner, Tamar Yablonski-Peretz, Shiri Shkedi-Rafid

**Affiliations:** 1 Sharett Institute of Oncology, Hadassah University Medical Center, Jerusalem, Israel; 2 Department of Genetics, Hadassah University Medical Center, Jerusalem, Israel; 3 Faculty of Medicine, The Hebrew University of Jerusalem, Jerusalem, Israel; 4 Department of Pathology, Hadassah University Medical Center, Jerusalem, Israel

**Keywords:** *TP53*, p.Arg181Cys, Arab-Muslim, Li-Fraumeni, Hereditary cancer syndrome

## Abstract

*TP53* pathogenic variants cause Li-Fraumeni syndrome (LFS), with some variants causing an attenuated phenotype. Herein, we describe the clinical phenotype and genetic characteristics of carriers of NM_000546.6 (*TP53*): c.541C > T, (p.Arg181Cys) treated at Hadassah Medical Center. We retrospectively examined our genetic databases to identify all carriers of *TP53* p.Arg181Cys. We reached out to carriers and their relatives and collected clinical and demographic data, lifestyle factors, carcinogenic exposures as well as additional blood samples for genetic testing and whole exome sequencing. Between 2005 and 2022 a total of 2875 cancer patients underwent genetic testing using genetic panels, whole exome sequencing or targeted *TP53* assays. A total of 30 cancer patients, all of Arab-Muslim descent, were found to be carriers of *TP53* p.Arg181Cys, the majority from Jerusalem and Hebron, two of which were homozygous for the variant. Carriers were from 24 distinct families of them, 15 families (62.5%) met updated Chompret criteria for LFS. Median age of diagnosis was 35 years-old (range 1–69) with cancers characteristic of LFS (16 Breast cancer; 6 primary CNS tumors; 3 sarcomas) including 4 children with choroid plexus carcinoma, medulloblastoma, or glioblastoma. A total of 21 healthy carriers of *TP53* p.Arg181Cys were identified at a median age of 39 years-old (range 2–54)—19 relatives and 2 additional pediatric non-cancer patients, in which the finding was incidental. We report a shared haplotype of 350kb among carriers, limited co-morbidities and low BMI in both cancer patients and healthy carriers. There were no demographic factors or carcinogenic exposures unique to carriers who developed malignancy. Upon exome analysis no other known pathogenic variants in cancer predisposing genes were identified. *TP53* p.Arg181Cys is a founder pathogenic variant predominant to the Arab-Muslim population in Jerusalem and Hebron, causing attenuated-LFS. We suggest strict surveillance in established carriers and encourage referral to genetic testing for all cancer patients of Arab-Muslim descent in this region with LFS-associated malignancies as well as family members of established carriers.

**Supplementary Information:**

The online version contains supplementary material available at 10.1007/s10689-024-00391-2.

## Introduction

*TP53* is a tumor suppressor regulating cell cycle, DNA repair, metabolism and apoptosis in response to DNA or cellular damage. Somatic variants in *TP53* are found in over 50% of all human cancers [1]. Germline pathogenic variants in *TP53* classically cause Li-Fraumeni syndrome (LFS) with most carriers developing characteristic cancers, for example adrenocortical carcinoma, sarcomas and primary central nervous system (CNS) tumors [2]. Testing for *TP53* is recommended for all patients who meet clinical LFS criteria, updated ‘Chompret Criteria’ (detailed in Table 2) [3, 4] and current surveillance recommendations for carriers include annual whole body and brain magnetic resonance imaging (MRI), biannual abdominal ultrasound from age of birth, and colonoscopy every 2 years from the age of 25 (the full surveillance protocol which is recommended in Israel is detailed in supplementary appendix 1) [3, 5, 6].

In recent years, a growing number of large cohorts have shown cancer patients found to be carriers of *TP53* pathogenic or likely pathogenic variants, who did not present with classical features of LFS, had late onset of cancer or cancers not previously associated with LFS and little or no family history of cancer [7–9]. The variability in the age of onset and penetrance was found to be correlated with specific variants in *TP53* [4, 10]. Yet, it is unclear whether other modifier genes or possibly additional environmental and lifestyle factors, including smoking or exposure to ionizing radiation, contribute to cancer development among carriers of these lower penetrance variants [11–13]. The clinical phenotype of attenuated-LFS has not been well described, and as such the appropriateness of the rigorous screening regimen recommended for classic LFS has not been proven in cases of attenuated LFS variants. If and how the surveillance should be modified in accordance with the expected milder phenotype, has yet to be formalized.

Israel is an ethnically diverse country, composed of a Jewish majority group (Ashkenazi and non-Ashkenazi) and an Arab minority group, commonly divided according to religion (Arab-Muslim, Arab-Christian and others). These communities rarely intermarry and are considered ethnically distinct. Since 2007 the annual incidence of malignancy in the Jewish population in Israel is steadily decreasing, while remaining unchanged in the Arab population, and even increasing among Arab women before the age of 35 [14–16]. Variants causing hereditary cancer syndromes are only partially described in the Arab population in Israel and world-wide, mainly variants in the *BRCA1/2* genes [15, 17, 18].

In a previous study we tested DNA samples from peripheral blood of cancer patients treated at Hadassah Medical Center (HMC) using a panel of 22 cancer predisposing genes. Of the 93 patients of Arab-Muslim descent which were tested, 7 patients (7.5%) were found to carriers of the variant NM_000546.6 (*TP53*): c.541C > T, (p.Arg181Cys) (chr17-7578389 G > A). These patients had LFS-associated cancers, varied familial history of cancer, though only four patients were diagnosed before the age of 50. This variant was not found in cancer patients of Arab-Christian or Jewish descent [19]. In a second cohort published by Hamaeh et al. which examined 453 patients with early onset or familial breast cancer of Arab descent from the Jerusalem area, The West bank and Gaza, the same *TP53*, p.Arg181Cys variant was found in 9 breast cancer probands (1.9%) in the Jerusalem area and lead to cascade detection of the variant in an additional 5 breast cancer patients and 5 healthy carriers [20].

The *TP53* p.Arg181Cys variant has not yet been classified by the ClinGen *TP53* Variant Curation Expert Panel and fulfils only partial criteria for pathogenicity according to the ACMG/AMP variant interpretation guidelines for germline *TP53* variants (PS4, PM2_Supporting, PM5_Supporting, PP3_Moderate, PS3_Supporting, BS2) [21, 22]. The variant has five submissions to the ClinVar database, from 2021 to 2023, by five different labs. The earliest submission (2021), based on the individual lab’s classification scheme was class 3 (uncertain significance). All other submissions, done in 2023, are class 4 and 5 (likely pathogenic or pathogenic). Two of these were based on ACMG criteria [23] and the other two were based on the labs’ classification schemes. Further search of The *TP53* database together with ClinVar and the gnomAD and FLOSSIES databases has revealed this variant in a total of 39 carriers of whom, 25 of known Arab-Muslim descent from the Jerusalem and Hebron area as detailed in the cohorts above, and the remaining 14 patients from Northern America and China. These comprise of 25 breast cancer patients, 9 healthy carriers and 5 cancer patients with LFS-associated tumors, including a pediatric patient with multiple tumors (rhabdomyosarcoma, adrenocortical carcinoma and osteosarcoma) [6, 24–30]. Further regarding characteristics of carriers of *TP53* p.Arg181Cys carriers described so far in the literature are provided in supplementary Table 1. In addition, a recent review of hereditary cancer syndromes in several Arab countries as well as targeted genetic testing conducted by Zidan et al. in cancer patients of Arab descent, suspected of heredity cancer syndrome in a different region in Israel did identify this variant [15, 17]. Finally, this variant was not identified in 282 healthy individuals from different 8 centers in Israel, genetically screened due to high-risk of breast or ovarian cancer [31].

The cumulative evidence to date, suggests that *TP53* p.Arg181Cys causes attenuated-LFS and is predominant to the Arab-Muslim population in the Jerusalem and Hebron area. However, the full clinical phenotype of this specific variant remains unknown, and it is not clear whether other demographic, clinical or genetic factors influence cancer susceptibility among carriers. Presently at HMC, identified carriers of the *TP53*, p.Arg181Cys variant are referred to a specialized carriership clinic for further genetic counseling. However, the screening and health surveillance recommendations for carriers has not yet been determined and currently carriers are advised to maintain strict surveillance protocols. This study further describes the clinical phenotype and genetic characteristics of carriers of *TP53* p.Arg181Cys carriers treated at Hadassah Medical Center**.**

## Materials and methods

### Participants

We retrospectively examined genetic databases for all cancer patients treated at HMC between 2005 and 2022, who underwent genetic testing using whole exome sequencing panel (WES), custom or commercial next-generation sequencing genetic panels (NGS) or targeted analysis of the *TP53* p.Arg181Cys variant using polymerase chain reaction assay (PCR) and were found to be carriers of *TP53* p.Arg181Cys. We reached out to carriers and their relatives and offered them the opportunity to enroll in further evaluation and genetic testing as part of our study.

### Clinical data

Clinical data was obtained from HMC electronic medical records, telemedicine and outpatient visits. These included clinical and demographic data, three-generation pedigrees, lifestyle factors (smoking, exercise, alcohol and drug use, sleeping hours and dietary habits), co-morbidities and environmental carcinogenic exposures (ionizing radiation, hormonal exposure etc. according to abbreviated list published by international agency for research on cancer [32]).

### Germline sample collection and analysis

For cancer patients and family members who were originally identified using *TP53* p.Arg181Cys PCR, additional WES was performed on collected DNA from peripheral blood samples using Ilumina Nextera Flex for enrichment kit (Illumina, San Diego, CA, USA) TM IDT xGen Exome Research Panel V2.0 capture (Integrated DNA Technologies, IA, USA) TM, and sequenced to a median depth of 80X using the NovaSeq 6000 sequencing system (Illumina, San Diego, CA, USA) TM as 100-bp paired-end runs.

### Haplotype analysis

A homozygous patient for the *TP53* p.Arg181Cys variant was initially identified from WES genetic testing results. These samples were used to establish a minimal homozygous region in the relevant chromosomal region of *TP53*, which was determined as the obligatory haplotype. A comparison of the conserved region on *TP53* to sequenced results from the additional *TP53* p.Arg181Cys carriers was conducted [33].

### Ethics

The study was authorized by HMC institutional IRB (0346-12-HMO) and by the IRB of the Israeli Health Ministry. All participants, probands and relatives consented to participation in a genetic study, including genetic testing and analysis. Healthy carriers identified as part and of the study, were referred to our high-risk carrier clinic for further cascade genetic screening of family members and surveillance in accordance with conventional LFS screening and surveillance recommendations.

## Results

### Genetic testing and identification of carriers of *TP53* p.Arg181Cys

Between 2005 and 2022 a total of 10,665 cancer patients were referred for genetic testing at HMC. Of them, 2875 patients were tested using WES or genetic panels or targeted PCR of *TP53* p.Arg181Cys based on known familial carriership or high clinical suspicion. These included two previously diagnosed cancer patients who were referred for genetic tested using targeted *TP53* p.Arg181Cys PCR, as a result of an incidental finding of the variant in two pediatric non-cancer probands (ages 4 and 18), who underwent WES due to developmental delay, and consequent cascade testing of their relatives (based on ACMG recommendations for reporting of secondary findings from WES [23]).

**Fig. 1:**
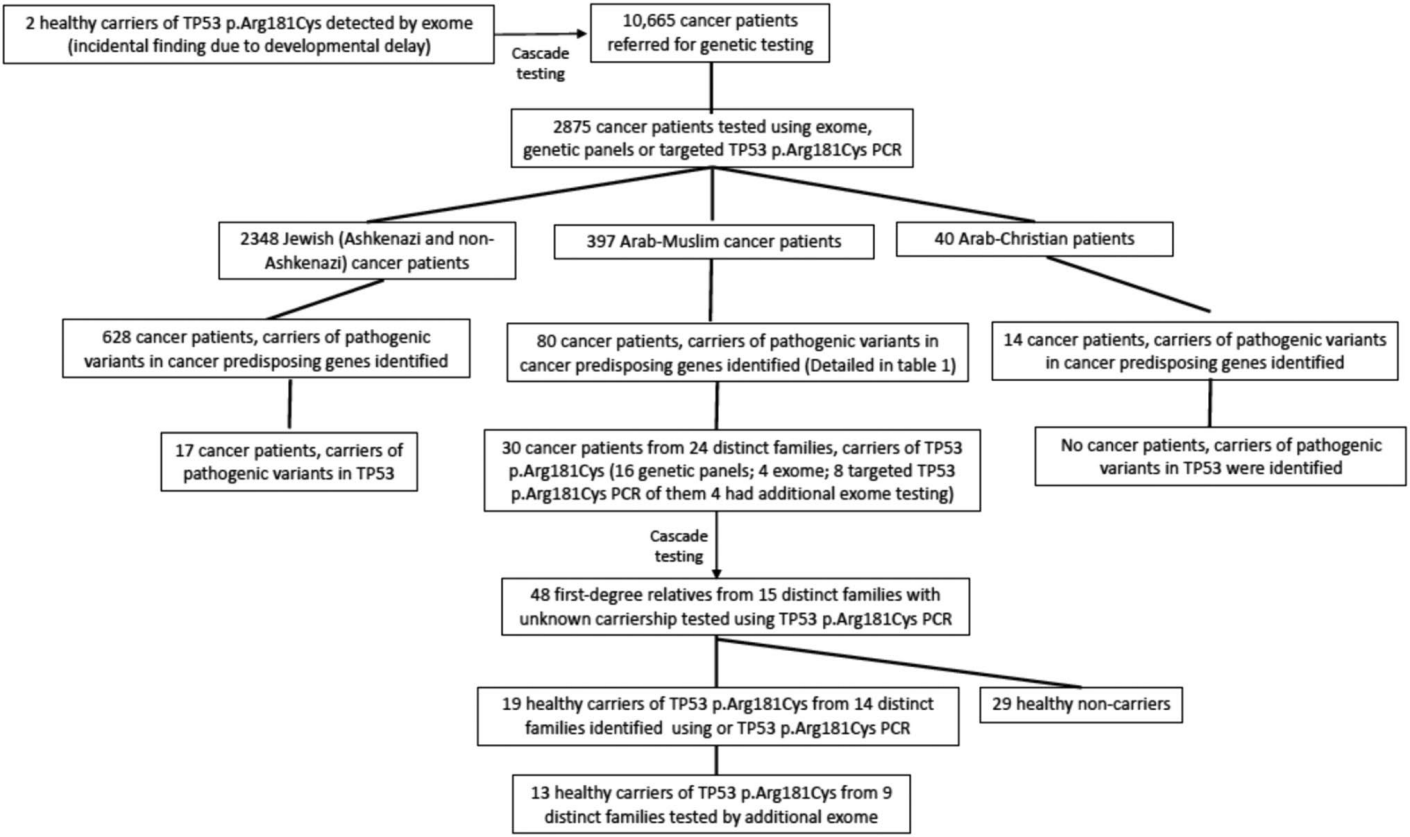
Flow chart of genetic testing conducted between 2005 and 2022 at Hadassah Medical Center and identification of carriers of *TP53* p.Arg181Cys variant

Of the 2875 cancer patients tested using relevant assays, 2438 were of Jewish descent (Ashkenazi and non-Ashkenazi), 397 were of Arab-Muslim descent and 40 were of Arab-Christian descent. Of the Arab-Muslim population, 80 patients (20.1%) were found to be carriers of pathogenic variants in cancer predisposition genes (Fig. 1; Table 1).

**Table 1:** Basic characteristics of cancer patients of Arab-Muslim descent treated at Hadassah Medical Center between 2005 and 2022 who were referred for genetic testing

Characteristic	N = 397 (%)
Age at diagnosis	
Median (Range)	46 (1–84)
Gender	
Female	285 (71.8)
Male	112 (28.2)
Cancer type	
Breast cancer	176 (44.3)
Colorectal cancer	61 (15.4)
Sarcoma	18 (4.5)
Thyroid cancer	17 (4.3)
Ovarian cancer	16 (4.0)
High grade CNS	16 (4.0)
Uterine	15 (3.8)
Lung cancer	10 (2.5)
Pancreas	8 (2.0)
Testicular cancer	5 (1.2)
Leukemia and lymphoma	3 (0.7)
Urothelial carcinoma	3 (0.7)
Head and neck	3 (0.7)
Adrenocortical cancer	1 (0.2)
Others or unknown	45 (11.3)
Pathogenic variants in major cancer predisposition genes	
Positive	80 (20.1)
*TP53* p.Arg181Cys	30 (7.6)
*BRCA 1* or* BRCA 2*	12 (3.0)
*APC*	12 (3.0)
*CFTR*	8 (2.0)
*ATM, FANCA, CHEK2, BRIP1, BARD1, MUTYH*	7 (1.7)
*MLH 1, MSH6, MSH2 or PMS2*	5 (1.2)
*SDHC, SDHA, LZTR1, NBN*	4 (1.0)
*RB1*	2 (0.5)
Negative	317 (79.9)

A total of 30 cancer patients, all of Arab-Muslim descent, were found to be carriers of the *TP53* p.Arg181Cys variant—16 patients were identified using NGS panels, 8 patients using targeted *TP53* p.Arg181Cys PCR based on cascade testing and 4 patients using WES. In four cancer patients initially identified using targeted *TP53* p.Arg181Cys PCR, additional WES was conducted. These 30 patients include the seven cancer patients previously described in the published cohort from HMC (supplementary Table 1) [19]. The *TP53* p.Arg181Cys variant was not found in any Jewish or Arab-Christian cancer patients tested using relevant assays. Additionally, no other germline variants in the *TP53* gene were found in the entire cohort of Arab-Muslim patients.

**Fig. 2:**
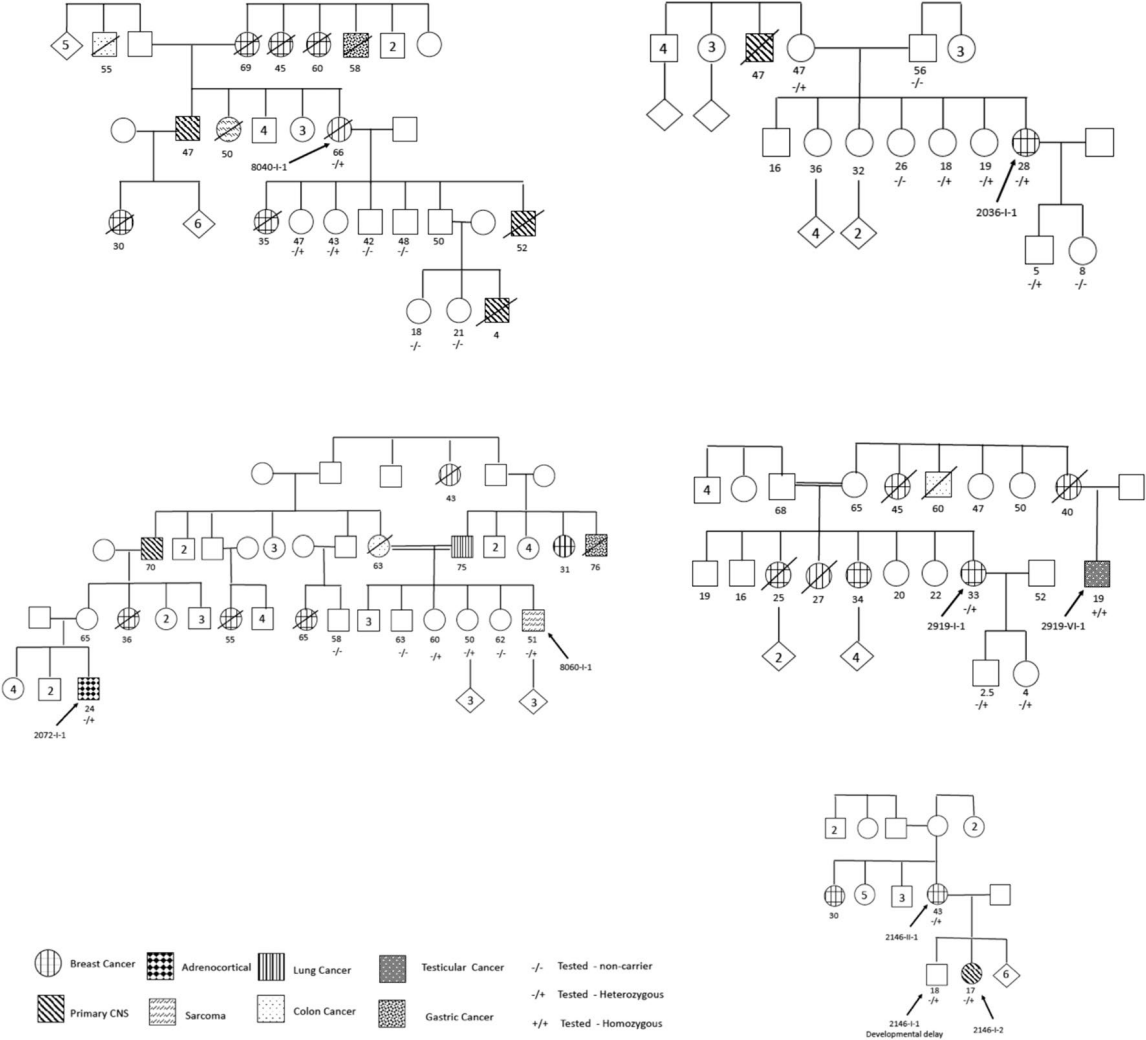
Examples of five pedigrees including six probands (black arrow) of carriers of *TP53* p.Arg181Cys showing variable family history of malignancy. Ages listed are age at cancer diagnosis or age of last follow up in case of healthy carrier

Cancer patients were of 24 distinct families (based on three-generation pedigrees), the majority of which originated from the Hebron (14 families) and Jerusalem (7 families) area. Of note, two probands (2072-I-1 and 8060-I-1) who were not known to be related and referred independently for genetic testing and consultation, were subsequently found to be related and were regarded as one family. Cancer patients reported significant yet variable family histories of malignancy with 20 families (83.3%) having at least one other first-degree or two second-degree relatives diagnosed with cancer. Of the 24 families, 15 (62.5%) met updated Chompret criteria for LFS (Table 2). Examples of family pedigrees are shown in Fig. 2.

**Table 2:** Basic characteristics of cancer patients, of Arab-Muslim descent carriers of *TP53* p.Arg181Cys treated at Hadassah Medical Center between 2005 and 2022

Familial characteristic	N = 24
Family history of malignancy^a^	
Positive	20 (83.3)
None	4 (16.7)
Meets updated criteria for Li-Fraumeni syndrome^b^	
Yes	15 (62.5%)
No	9 (37.5%)
Familial origin—according to family	
Hebron	14 (58.3)
Jerusalem	7 (29.2)
Turkey	2 (8.3)
Morocco	1 (4.2)

Personal characteristic	N = 30
Age at diagnosis	
Median (Range)	35 (1–69)
Over 50 years old	4 (13.3)
Under 18 years old	4 (13.3)
Gender	
Female	21 (70.0)
Male	9 (30.0)
Cancer type	
Breast cancer	16 (53.3)
HER2—positive	6 (20)
HER2—low and ER-positive	2 (6.6)
HER2—negative and ER-positive	8 (26.7)
Primary high grade CNS tumor	6 (20.0)
Glioblastoma	3 (10.0)
Choroid plexus	2 (6.6)
Medulloblastoma	1 (3.3)
Sarcoma	3 (10)
Liver: epithelioid hemangioendothelioma	1 (3.3)
Lower limb: osteosarcoma	1 (3.3)
Head and neck: leiomyosarcoma	1 (3.3)
Testicular	2 (6.6)
Adrenocortical and lymphoma	1 (3.3)
Lung	1 (3.3)
Head and neck: squamous cell	1 (3.3)
Stage at diagnosis	
Stage I—local disease	9 (30.0)
Stage II–III—locally advanced	11 (36.7)
Stage—IV—metastatic	4 (13.3)
Primary high grade CNS tumor	6 (20.0)
Co-morbidities	
None	26 (86.7)
Yes	4 (13.3)
Ischemic heart disease	2 (6.6)
Diabetes	2 (6.6)
Body mass index (26 patients)	24.5 (range 21.9–31.9)

^a^Familial history—At least one 1st degree relative or two 2nd degree relatives with malignancy typical of Li-Fraumeni syndrome

^b^According to updated Chompret Criteria [3, 4]: A Li-Fraumeni syndrome (LFS)-related tumor, before the age of 46 and at least one first-degree or second-degree family member with an LFS-related tumor (except breast cancer if the proband has breast cancer) before the age of 56 or with multiple tumors; or Multiple tumors, except multiple breast tumors, two of which belong to the LFS tumor spectrum, and first of which occurred, irrespective of family history; or Patient with adrenocortical carcinoma, choroid plexus carcinoma, or rhabdomyosarcoma of embryonal anaplastic subtype, irrespective of family history; or Breast cancer before 31 years, irrespective of family history

In all cancer patients who underwent WES or NGS panels (a total of 26 of 30 patients, 86.7%) no other pathogenic or likely pathogenic variants in cancer predisposing genes were found (based on ClinGen Expert Panel curation and ACMG guidelines [23, 34]). Variants of unknown clinical significance were found in a few cancer patients, detailed in supplementary Table 2. Of the 30 cancer patients, 2 were homozygous for *TP53* p.Arg181Cys—a 23-year-old female patient diagnosed with HER2-positive metastatic positive breast cancer (3577-I-1/ Zic267-I-1) and a 19-year-old male diagnosed with stage I testicular cancer (2919-VI-1).

### Familial genetic testing and haplotype analyses of carriers of *TP53* p.Arg181Cys

A total of 48 first-degree relatives from 15 different families were tested using targeted *TP53* p.Arg181Cys PCR. Of them, 19 (39.6.0%) from 14 different families were found to be healthy carriers of TP*53* p.Arg181Cys, in addition to the two already established carriers detected due to the incidental finding in children with developmental delay. Median age of the 21 healthy carriers was 39 years old (range 2–54). The remaining 29 family members were non-carriers and had no history of malignancy, except for one patient with a history of hepatocellular carcinoma (supplementary Table 1). In 13 of 21 healthy carriers (61.9%) identified, WES was conducted with no additional findings in cancer predisposing genes (based on ClinGen expert panel curation and ACMG guidelines [23, 34]).

**Fig. 3:**
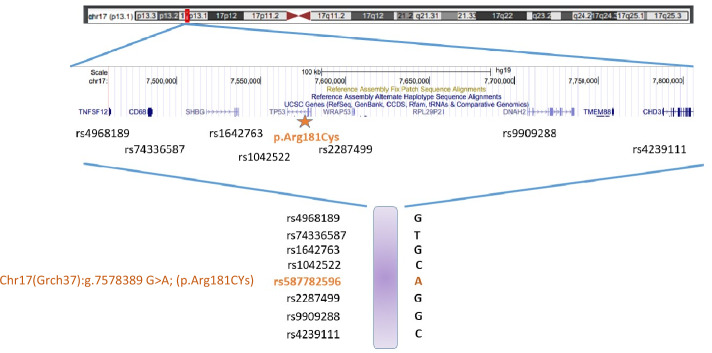
Haplotype analysis of *TP53* p.Arg181Cys. Using whole exome sequencing on DNA sample of one patient homozygous for the *TP53* p.Arg181Cys variant we identified a mutual homozygous region in the relevant chromosomal region of TP53 spanning at least 350kb which was determined as the obligatory haplotype. *TP53* sequenced results from the additional *TP53* p.Arg181Cys carriers (8 cancer patients from 8 families and 13 healthy carriers from 9 families) found this region to be shared among all carriers tested. The locations of the 7 SNPs comprising the haplotype depicted are outlined below: rs4968189:Chr17(GRCh37):g.7460559 T > G; rs74336587: Chr17(GRCh37):g.7489417 C > T; rs1642763: Chr17(GRCh37):g.7557419 A > G; rs1042522: Chr17(GRCh37):g.7579472 G > C located on the p.Arg72 allele of *TP53*; rs2287499: Chr17(GRCh37):g.7592168 C > G; rs9909288: Chr17(GRCh37):g.7673928 C > G; rs4239111: Chr17(GRCh37):g.7811998 T > C

WES results of the homozygous patient 3577-I-1/ Zic267-I-1 enabled to determine a minimal homozygous region in the chromosomal area of *TP53* (codding regions) which corresponded to a minimal shared haplotype for *TP53* p.Arg181Cys spanning at least 350-kb. This haplotype was conserved among all carriers in which blood samples were available for WES analysis (8 cancer patients from 8 families and 13 healthy carriers from 9 families), and included the single nucleotide polymorphism (SNP) rs1042522: Chr17(GRCh37), (p.Arg72). (Figs. 1, 3).

### Clinical characteristics of cancer patients carriers of *TP53* p.Arg181Cys

Median age of diagnosis for the 30 cancer patients was 35 years-old (range 1–69), with high variability, including four children (13.3%) with high-grade primary CNS tumors—choroid plexus carcinoma at 1 and 4 years-old, medulloblastoma at 2 years-old, and glioblastoma at 17 years-old. Only 5 patients (16.7%) were diagnosed with cancer over the age of 50. The types of cancers diagnosed were characteristic of LFS and included breast cancer—16 patients, (53.3%, of them, 6 patients were HER2-positive 2 patients were HER2-low and 3 patients were diagnosed before the age of 31); primary high-grade CNS tumors—6 patients (20%); and sarcomas—3 patients (10.0%). Only 16 cancer patients (53.3%) met updated Chompret Criteria for LFS [3, 4]. Clinical outcomes for cancer patients were relatively favorable, including median overall survival of 30 months (range 27–34) for the 3 patients diagnosed with glioblastoma and 44 months (range 18–52) for 4 patients treated for metastatic breast cancer. In addition, only 2 of 8 patients who were diagnosed with locally advanced breast cancer (stages IIB-IIIB) suffered disease recurrence after definitive treatment. Notably, two breast cancer patients were diagnosed with stage I breast cancer and underwent curative surgery, as a result of cascade genetic testing in and subsequent referral for early screening. (Clinical characteristics of cancer patients are detailed in Table 2 and supplementary Table 1).

A review of medical records and interview of healthy carriers, cancer patients and their families, including examination of demographic, lifestyle factors and carcinogenic exposures (smoking, ionizing radiation, hormonal exposure etc.) showed no unique characteristics in carriers who developed malignancy compared to healthy carriers. Notably, only 4 cancer patients (13.3%) and 2 adult healthy carriers (12.5%) had additional co-morbidities, mainly ischemic heart disease, and diabetes. In addition, both adult cancer patients and adult healthy carriers had on average low body-mass index (BMI) with a median of 24.5 (range 21.9–31.9) and 22.1 (range 21.0–29.6), respectively, with only four cancer patients and two healthy-carriers with BMI of over 30.

## Discussion

We present here the clinical phenotype, demographic and genetic characteristics of carriers of the *TP53*, p.Arg181Cys variant of Arab-Muslim descent treated at our center with a focus on 30 cancer patients and their corresponding pedigrees. The combination of the cancer types, variable age at onset, and discrepancy in family history of malignancy, with 15 of 24 families meeting updated Chompret criteria for LFS, supports a decreased penetrance and an attenuated phenotype for this *TP53* variant. Nevertheless, the majority of cancer patients (25 of 30) in this cohort were diagnosed before the age of 50 including four carriers who were diagnosed with primary CNS tumors in childhood. Furthermore, no additional familial, demographic, environmental or genetic factors were found that may explain the differences in age of onset of cancer. The lack of other factors which can assist in the prediction of the degree of penetrance amongst carriers is in-line with previous analyses conducted on carriers of attenuated *TP53* variants [13]. We therefore recommend that carriers of *TP53* p.Arg181Cys follow strict surveillance and early detection tests akin to those recommended in classical LFS, specifically the use of annual whole body and brain MRI from infancy. Future analysis of genetic modifiers, somatic alterations, and clinical data and by age group, may allow for a more accurate understanding of age-associated risks, which could ultimately inform age-adjusted surveillance protocols.

Considering that the *TP53* p.Arg181Cys variant was found exclusively in the Arab-Muslim population, the majority of which originated from of the Jerusalem or Hebron area, the shared haplotype, together with the fact that this variant has rarely been described outside this geographical area, we suggest that *TP53* p.Arg181Cys is a founder mutation predominant to the Arab-Muslim population in this area. While attenuated LFS is well described in the context of ever-increasing genetic testing in cancer patients, the finding of a pathogenic variant causing an attenuated phenotype together with a founder effect in large number of carriers (30 cancer patients and 21 healthy carriers) isolated to a specific region and population, has been rarely described outside the Brazilian and Jewish-Ashkenazi variant [35, 36]. In light of its increased frequency among the sub-population of Arab patients in Israel, *TP53* p.Arg181Cys was included in 2020 as one of the pathogenic variants tested as part of the founder mutations panel recommended by the Israeli Ministry of Health to all Arab-Muslim breast cancer patients, regardless of their age at diagnosis. We suggest broadening this recommendation to all patients of Arab-Muslim descent diagnosed with cancer characteristic of LFS of, as well as reaching out to family members of known carriers of *TP53* p.Arg181Cys for cascade genetic testing. Healthy carriers identified can then be recommended the appropriate surveillance regimen. Such cascade testing and subsequent screening has already benefited at least two patients in this cohort diagnosed with early-stage breast cancer as a result of incidental identification of the variant in non-cancer patients referred for genetic testing due to developmental delay.

Notably, *TP53* p.Arg181Cys has been described in one other pediatric case—a 1 year old boy with rhabdomyosarcoma, who was later diagnosed with adrenocortical carcinoma and osteosarcoma at 2 years of age, as a result of whole body MRI surveillance, demonstrating the importance of genetic screening for *TP53* p.Arg181Cys and subsequent surveillance in high-risk population [6]. This cohort is the first to report the *TP53* p.Arg181Cys variant in several very young pediatric cases, and numerous cases in the early adulthood, further warranting genetic screening of this this high-risk population and strict surveillance protocols for all established *TP53* p.Arg181Cys carriers.

The *TP53* p.Arg181Cys variant resembles the *TP53* p.Arg337His founder variant detected in one of every 300 individuals in Southeastern Brazil. Similar to our cohort, while initially reported in cases of pediatric adrenocortical carcinoma patients, large-scale studies have since described breast cancer as the most common tumor in carriers of *TP53* p.Arg337His with later onset in comparison to classical LFS [35, 37]. Additionally, p.Arg337His confers a highly variable cancer risk, ranging from individuals who remain unaffected over their lifetime to those who meet the Chompret Criteria for LFS. This variability was not found to be influenced by demographic factors or carcinogenic exposures but rather mediated by the co-inheritance other pro-apoptotic tumor suppressors, such as XAF1 p.E134^*^ [35]. Finally, current surveillance and screening recommendation for *TP53* p.Arg337His are in line with those suggested for classical LFS and have found to be both efficient in reducing cancer mortality and cost-effective [38].

The *TP53* p.Arg181Cys variant also resembles the *TP53* p.Gly334Arg variant reported in 22 cancer patients from 16 families predominantly of Jewish-Ashkenazi descent in Northern America. Though 4 of 22 cancer patients were diagnosed with pediatric adrenocortical carcinoma, the most common tumor among carriers of *TP53* p.Gly334Arg was breast cancer (10 of 22 patients) with relatively late onset (range 30–65 years old). Additionally, while most probands reported a family history of cancer, only 6 of 16 probands in which ancestry was available met updated Chompret Criteria for LFS thus, providing evidence of reduced penetrance in a *TP53* variant distinct to a specific population [36].

The fact that two young cancer patient in our cohort are homozygous for *TP53* p.Arg181Cys is intriguing and supports the partial oncogenic properties of this variant. While over 90% of carriers of *TP53* who develop LFS-associated tumors, exhibit somatic loss of the wild-type allele [39], germline bi-allelic variations, including homozygosity, have so far been described in only a handful of cases. Most of these were in carriers of the Brazilian variant, and all presented with early onset of cancer, albeit limited penetrance among their family members [40–42]. While a recent haplotype analysis of 38 unrelated carriers of *TP53* p.Arg337His found two homozygous patients with early onset of cancer (ages 6 and 9 years old) with a compound *TP53* p.Arg337His and XAF1 p.E134^*^ haplotype, other reports did not rule out additional co-variants[43]. WES done on one homozygous patient in our cohort did not identify additional variations in known *TP53* modifiers. Hence, it remains unclear if homozygosity, or rather the presence of other co-variants leads to increased penetrance among these homozygous patients.

The *TP53* 181 codon is a H1 helix residue is located in the apoptosis-stimulating of P53 protein 2-domain (ASPP2). While structural models have shown this codon to be essential for dimer stability, modification of *TP53* 181 retains partial protein function. *TP53* p.Arg181 modifications in cancer-cell lines and mouse models have shown to be able to activate p21CDKN1A or MDM2 at levels similar to wild-type *TP53*, but unable to activate genes associated with apoptosis, such as NOXA or p53AIP1 [44, 45]. *TP53* p.Arg181Cys in mice demonstrated a modest increase in cancer incidence and increased lipolytic activity implicated in cancer development [46]. Several functional analyses of an array of *TP53* variants expressed in yeast or mammalian cells, which were recently shown to be highly correlated, determine *TP53* p.Arg181Cys to be an outlier variant with partial loss of apoptotic activity. These assays also demonstrate that while *TP53* p.Arg181Cys loses some tumor suppressor functions, it does not cause additional oncogenic gain of function (GOF) which is typical to *TP53* variant ‘hotspots’ associated with classical LFS [47–49]. Recently, Landau et al. implemented a more continuous computational approach to determine *TP53* pathogenicity, using tumor variant amplitude, in which *TP53* p.Arg181Cys was shown be a weak oncogenic driver [50]. Finally, analyses of other variants causing attenuated LFS, mainly the Brazilian variant, have shown cancer susceptibility to be dependent on co-inheritance of other proapoptotic tumor suppressors involved in *TP53* mechanism [35]. These animal models and functional and computational analyses may explain the pathogenicity and reduced penetrance of *TP53* p.Arg181Cys, demonstrated in our clinical findings.

Cancer patients in our cohort had relatively good clinical outcomes. While *TP53* mutations have shown mixed impact on clinical outcomes in cases of breast cancer, *TP53* mutations are surrogate for markedly improved prognosis in CNS tumors [51, 52]. As previously mentioned, *TP53* p.Arg181Cys has limited penetrance and a phenotype resembling attenuated-LFS, by retention of several wild-type properties which relegate it a weak oncogene driver when compared to pathogenic variants in hotspots causing classical LFS. Previous studies, both clinical and computational, have shown a correlation between the degree of penetrance of attenuated-LFS and survival [4, 7, 50].

Interestingly, both cancer patients and healthy carriers had limited co-morbidities, were in good general health and had low BMI. These findings hint to potentially favorable properties of *TP53* p.Arg181Cys, which may contribute to the good clinical outcomes. A previous in-vivo analysis of muscle specimens taken from volunteers after exercise, demonstrate that healthy carriers of *TP53* variants, have better muscle recovery, improved recovery of phosphocreatine and enhanced mitochondrial function compared to healthy non-carriers. This was substantially more prominent for the 7 carriers of *TP53* p.Arg181Cys included in the study, in which levels of mitochondrial respiratory complex proteins associated with biogenesis were significantly increased [25]. *TP53* p.Arg181Cys knock-in mice show a uniquely high proportion of lean muscle tissue, increased aerobic exercise endurance, increased lipolytic activity and transactivation of genes involved in fatty-acid metabolism which do not appear in knock-in models of other *TP53* variants [46]. In addition, haplotype analysis conducted in this cohort show that *TP53* p.Arg181Cys appeared in conjunction with the p.Arg72 SNP in both cancer patients and healthy carriers. While this SNP has limited impact on cancer risk, data from cell lines and mice models show that this SNP markedly affects the response of P53 to nutrient alterations, driving increased inflammation in mice on a high-fat diet and alters the ability of mutant P53 to bind and inhibit the PGC-1α metabolism regulator thus inducing cancer-promoting metabolism [53]. Our clinical results support this in-vivo evidence that *TP53* p.Arg181Cys acts as a double edge sword – causing attenuated-LFS while at the same time enhancing lipolytic activity and metabolism which in turn may promote tumorigenesis. Moreover, these findings hint at the potential therapeutic approach to delay cancer development among *TP53* p.Arg181Cys carriers by decreasing mitochondrial function through common anti-diabetic medication, a theory based on mouse models [54]. Finally, analysis of other attenuated LFS variants in *TP53* have shown that cancer development is dependent on the development of compound mutant haplotypes including other pro-apoptotic tumor suppressors and loss of somatic wildtype *TP53* alleles [39, 43].

Given the rarity of *TP53* variants, our clinical data on 30 cancer patients carriers and 21 healthy carriers and their families due to a founder effect in a specific population and region, is highly valuable. Nevertheless, our study was limited since we could not complete genetic testing in all family members, nor build more detailed age-specific pedigrees is some families, which could have provided more informative data for age-associated penetrance. In addition, some of the healthy carriers identified are still young and therefore may develop cancer in the future, which will impact the ratio of affected to unaffected carriers. Long-term data should be collected from these families as the carriers age and the families expand, to further determine our findings over time and perhaps lead to a less rigid surveillance protocol.

## Conclusion

Our findings suggests that *TP53* p.Arg181Cys is a founder pathogenic variant predominant to the Arab-Muslim population in the Jerusalem and Hebron area, causing attenuated-LFS. While this variant has shown limited penetrance, the high prevalence of young cancer patients in our cohort, including 4 pediatric patients with CNS tumors, and lack of demographic, environmental or other modifiers that could account for the phenotypic heterogeneity, suggests that early and strict detection tests and surveillance should be continued for known carriers of *TP53* p.Arg181Cys, akin to those recommended in classical LFS. We further encourage awareness and referral to genetic testing of all cancer patients of Arab-Muslim descent in this region, in particular those diagnosed with malignancies associated with LFS, regardless of age at diagnosis and family history. We encourage cascade testing among healthy family members of known carriers. Further analysis of genetic modifiers, somatic alterations, and clinical data according to age groups may allow a more accurate understanding of the limited penetrance and mechanisms of cancer development caused by this variant, which could ultimately inform more tailored surveillance and screening protocols.

## Supplementary Information

Below is the link to the electronic supplementary material.

Supplementary file1 (DOCX 60 KB)

## Data Availability

No datasets were generated or analysed during the current study.
