# Supplementary material for: Clinical and genetic characteristics of carriers of the TP53 c.541C > T, p.Arg181Cys pathogenic variant causing hereditary cancer in patients of Arab-Muslim descent
Source: Fam Cancer. 2024 May 14;23(4):531–42. doi: 10.1007/s10689-024-00391-2 (PMC11512851; doi:10.1007/s10689-024-00391-2)
Supplement: Supplementary file 1 — Supplementary file1 (DOCX 60 KB) [file 10689_2024_391_MOESM1_ESM.docx]

**Supplementary Table 1** – Basic characteristics of carriers of *TP53* p.Arg181Cys treated at Hadassah Medical Center and described so far in the literature according to a search of

- ClinVar (https://www.ncbi.nlm.nih.gov/clinvar/variation/142624)

- The *TP53* Database (https://tp53.isb-cgc.org)

- gnomAD (https://gnomad.broadinstitute.org/gene/ENSG00000141510)

- FLOSSIES (https://whi.color.com/gene/ENSG00000141510)

| Case Index * | Geographic Origin | Origin | Family Member | Age ** | Gender | Tumor Origin / Healthy Carrier | Chompret Criteria *** | Reference |
| --- | --- | --- | --- | --- | --- | --- | --- | --- |
| This cohort including previously published cohort by Zick et al [19] | | | | | | | | |
| 2919-I-1 and Zic-1244-I-1 | Hebron | Arab-Muslim | Proband | 33 | F | Breast: ER+ HER2- | Yes | ^ and [19] |
| 2919-VI-1 | Hebron | Arab-Muslim | Relative | 19 | M | Testicular: Seminoma  * Homozygous* | Yes | ^ |
| 2828-I-1 and Zic-myr-I-1 | Turkey | Arab-Muslim | Proband | 49 | F | Breast: ER+ HER2+ | No | ^ and [19] |
| 8603-I-1 and Zic227-I-1 | Turkey | Arab-Muslim | Proband | 46 | M | Lung: Adenocarcinoma | No | ^ and [19] |
| 8040-I-1 and Zic242-I-1 | Hebron | Arab-Muslim | Proband | 66 | F | Breast: ER+ HER2+ | Yes | ^ and [19] |
| 8040-II-1 | Hebron | Arab-Muslim | Relative | 45 | F | Healthy Carrier | Yes | ^ |
| 8040-II-1 | Hebron | Arab-Muslim | Relative | 47 | F | Healthy Carrier | Yes | ^ |
| 3577-I-1 and Zic267-I-1 | Jerusalem | Arab-Muslim | Proband | 23 | F | Breast: ER-, HER2+  * Homozygous* | Yes | ^ and [19] |
| 3577-II-2 | Jerusalem | Arab-Muslim | Relative | 21 | F | Healthy Carrier | Yes | ^ |
| 4313-I-1 and ZIc521-I-1 | Hebron | Arab-Muslim | Proband | 27 | F | Breast: ER+, HER2- | Yes | ^ and [19] |
| 4313-II-1 and Zic521-II-1 | Hebron | Arab-Muslim | Relative | 69 | M | Brain: Glioblastoma | Yes | ^ and [19] |
| 3649-I-1 | Hebron | Arab-Muslim | Proband | 35 | F | Breast: ER+, HER2-(low) | No | ^ |
| 6675-I-1 | Hebron | Arab-Muslim | Proband | 28 | F | Breast: ER-, HER2- | Yes | ^ |
| 2592-I-1 | Jerusalem | Arab-Muslim | Proband | 42 | F | Breast: ER-, HER2+ | No | ^ |
| 2592-I-2 | Jerusalem | Arab-Muslim | Relative | 46 | F | Healthy Carrier | No | ^ |
| 2592-I-3 | Jerusalem | Arab-Muslim | Relative | 50 | F | Healthy Carrier | No | ^ |
| 6618-I-1 | Jerusalem | Arab-Muslim | Proband | 37 | F | Breast: ER+, HER2- | No | ^ |
| 8604-I-1 | Hebron | Arab-Muslim | Proband | 47 | F | Breast: ER-, HER2+ | No | ^ |
| 8604-II-1 | Hebron | Arab-Muslim | Relative | 24 | F | Healthy Carrier | No | ^ |
| 2072-I-1 | Jerusalem | Arab-Muslim | Proband | 24 | M | Adrenocortical and Lymphoma | Yes | ^ |
| 2036-I-1 | Hebron | Arab-Muslim | Proband | 28 | F | Breast: ER+, HER2- | Yes | ^ |
| 2036-II-1 | Hebron | Arab-Muslim | Relative | 49 | F | Healthy Carrier | Yes | ^ |
| 2036-I-2 | Hebron | Arab-Muslim | Relative | 19 | F | Healthy Carrier | Yes | ^ |
| 2036-I-3 | Hebron | Arab-Muslim | Relative | 18 | F | Healthy Carrier | Yes | ^ |
| 2047-I-1 | Hebron | Arab-Muslim | Proband | 27 | M | Liver: Epithelioid Hemangioendothelioma | Yes | ^ |
| 8060-I-1 | Hebron | Arab-Muslim | Proband | 51 | M | Head and Neck: Leiomyosarcoma | Yes | ^ |
| 8060-I-2 | Hebron | Arab-Muslim | Relative | 50 | F | Healthy Carrier | Yes | ^ |
| 8060-I-3 | Hebron | Arab-Muslim | Relative | 60 | M | Healthy Carrier | Yes | ^ |
| 8059-I-1 | Hebron | Arab-Muslim | Proband | 46 | M | Brain: Glioblastoma | Yes | ^ |
| 2007-I-1 | Morocco | Arab-Muslim | Proband | 33 | F | Breast: ER+, HER2-(low) | Yes | ^ |
| 2007-I-2 | Morocco | Arab-Muslim | Relative | 22 | M | Testicular: Seminoma | Yes | ^ |
| 2007-I-3 | Morocco | Arab-Muslim | Relative | 54 | F | Healthy Carrier | Yes | ^ |
| 2765-I-1 | Hebron | Arab-Muslim | Proband | 51 | M | Lower Limb: Osteosarcoma | No | ^ |
| 6621-I-1 | Hebron | Arab-Muslim | Proband | 38 | F | Head and Neck: Squamous Cell Carcinoma | Yes | ^ |
| 8026-I-1 | Hebron | Arab-Muslim | Proband | 68 | F | Breast: ER+, HER2- | No | ^ |
| 8026-I-2 | Hebron | Arab-Muslim | Relative | 69 | F | Breast: ER+, HER2- | No | ^ |
| 8026-I-3 | Hebron | Arab-Muslim | Relative | 48 | F | Healthy Carrier | No | ^ |
| 3404-I-1 | Hebron | Arab-Muslim | Proband | 18 | M | Healthy Carrier: Developmental delay | No | ^ |
| 3404-II-1 | Hebron | Arab-Muslim | Relative | 42 | F | Healthy Carrier | No | ^ |
| 3280-I-1 | Hebron | Arab-Muslim | Proband | 1 | F | Brain: Choroid Plexus | Yes | ^ |
| 3280-II-1 | Hebron | Arab-Muslim | Relative | 54 | M | Healthy Carrier | Yes | ^ |
| 3280-I-2 | Hebron | Arab-Muslim | Relative | 34 | F | Healthy Carrier | Yes | ^ |
| 3280-I-3 | Hebron | Arab-Muslim | Relative | 27 | M | Healthy Carrier | Yes | ^ |
| 3421-I-1 | Jerusalem | Arab-Muslim | Proband | 4 | F | Brain: Choroid Plexus | Yes | ^ |
| 2629-I-1 | Jerusalem | Arab-Muslim | Proband | 47 | F | Breast: ER+, HER2- | No | ^ |
| 2629-II-1 | Jerusalem | Arab-Muslim | Relative | 18 | M | Healthy Carrier | No | ^ |
| 2146-I-1 | Jerusalem | Arab-Muslim | Proband | 18 | F | Healthy Carrier:  Developmental delay | Yes | ^ |
| 2146-I-2 | Jerusalem | Arab-Muslim | Relative | 17 | F | Brain: Glioblastoma | Yes | ^ |
| 2146-II-1 | Jerusalem | Arab-Muslim | Relative | 43 | F | Breast: ER-, HER2+ | Yes | ^ |
| 2351-I-1 | Jerusalem | Arab-Muslim | Proband | 2 | F | Brain: Medulloblastoma | Yes | ^ |
| 2351-II-1 | Jerusalem | Arab-Muslim | Relative | 27 | M | Healthy Carrier | Yes | ^ |
| Hamameh et al. [20] | | | | | | | | |
| MK7II2 | Jerusalem | Arab-Muslim | Relative | 68 | F | Healthy Carrier | Yes | [20] |
| MK7II5 | Jerusalem | Arab-Muslim | Relative | 55 | F | Healthy Carrier | Yes | [20] |
| MK7III2 | Jerusalem | Arab-Muslim | Relative | 46 | M | Healthy Carrier | Yes | [20] |
| MK7III11 | Jerusalem | Arab-Muslim | Relative | 25 | F | Healthy Carrier | Yes | [20] |
| MK7II7 | Jerusalem | Arab-Muslim | Relative | 47 | F | Breast | Yes | [20] |
| MK7III6 | Jerusalem | Arab-Muslim | Proband | 32 | F | Breast | Yes | [20] |
| MK7III8 | Jerusalem | Arab-Muslin | Relative | 30 | F | Breast | Yes | [20] |
| MK7III10 | Jerusalem | Arab-Muslim | Relative | 29 | F | Breast | Yes | [20] |
| MK1GGI | Jerusalem | Arab-Muslim | Proband | 45 | F | Breast | Yes | [20] |
| MKLRI | Jerusalem | Arab-Muslim | Proband | 41 | F | Breast | No | [20] |
| MKYEII1 | Jerusalem | Arab-Muslim | Proband | 41 | F | Breast - multiple and Leukemia | Yes | [20] |
| MKYEIII2 | Jerusalem | Arab-Muslim | Relative | 38 | F | Healthy Carrier | Yes | [20] |
| MKYEIII1 | Jerusalem | Arab-Muslim | Relative | 41 | F | Breast | Yes | [20] |
| MK1FJI | Jerusalem | Arab-Muslim | Proband | 68 | F | Breast | Yes | [20] |
| MK8I | Jerusalem | Arab-Muslim | Proband | 41 | F | Breast | No | [20] |
| MK1FPI | Jerusalem | Arab-Muslim | Proband | 27 | F | Breast | Yes | [20] |
| MK1CLII1 | Jerusalem | Arab-Muslim | Proband | 60 | F | Breast | No | [20] |
| MKWEI1 | Jerusalem | Arab-Muslim | Proband | 39 | F | Breast | No | [20] |
| Wang et al [25] | | | | | | | | |
| WAN13-1-4 | Northern America | N/A | Proband | 48 | N/A | Melanoma and Renal Cell Carcinoma | No | [25] |
| WAN13-1-8 | Northern America | N/A | Relative | 43 | N/A | Breast | No | [25] |
| WAN13-1-1 | Northern America | N/A | Relative | 50 | N/A | Healthy Carrier | No | [25] |
| WAN13-1-5 | Northern America | N/A | Relative | 60 | N/A | Healthy Carrier | No | [25] |
| WAN13-1-6 | Northern America | N/A | Relative | 38 | N/A | Healthy Carrier | No | [25] |
| WAN13-1-7 | Northern America | N/A | Relative | 50 | N/A | Healthy Carrier | No | [25] |
| WAN13-1-9 | Northern America | N/A | Relative | 19 | N/A | Healthy Carrier | No | [25] |
| Others | | | | | | | | |
| VIL-16-16-I | Northern America | N/A | Proband | 1 | M | Rhabdomyosarcoma, Adrenocortical Carcinoma and Osteosarcoma | Yes | [6] |
| KWON-F08-II | China | N/A | Proband | 36 | F | Breast | N/A | [27] |
| KWON-F08-II | China | N/A | Relative | 42 | F | Breast | N/A | [27] |
| SHEN15099 | China | N/A | Proband | 55 | F | Breast | N/A | [26] |
| OWS-137 | China | N/A | Proband | 43 | F | Breast | N/A | [30] |
| Side92III-2 | Northern America | Caucasian | Proband | 33 | F | Breast and Spindle Cell Sarcoma | Yes | [29] |
| HEK16-1-I-1 | Northern America | Caucasian | Proband | 80 | M | Pancreatic | N/A | [24] |

* According to the *TP53* database

** Age of first tumor onset or last follow up in cases of healthy carriers

*** According to Family Chompret Criteria

^ Described in current cohort

**Supplementary Table 2 -** Variants of unknown significance identified in whole exome sequencing or next generation sequencing panel in cancer patients and healthy carriers of the *TP53* p.Arg181Cys variant(based on ACMG criteria).

| Patient | Gene | HGVSc | HGVSp | dbSNP |
| --- | --- | --- | --- | --- |
| 2919-I-1 | NM_004448.4 *(ERBB2)* | c.1466C>T | p.Pro489Leu | rs142456637 |
|  | NM_000057.4 *(BLM)* | c.715G>C | p.Asp239His | rs200756519 |
| 3421-I-1 | *NM_001018115.3 (FANCD2)* | c.1098+4A>G | | rs664233 |
|  | *NM_000135.4 (FANCA)* | c.4260+6G>C | | rs374793201 |
| 4313-I-1 | *NM_032043.3 (BRIP1)* | c.2594G>A | p.Arg865Gln | rs781609846 |
|  | NM_004448.4 *(ERBB2)* | c.1466C>T | p.Pro489Leu | rs142456637 |
|  | *NM_177438.3 (DICER1)* | c.3382G>A | p.Val1128Ile | |
| 2592-II-1 | NM_000057.4  *(BLM)* | c.2555+5del |  |  |
|  | NM_001982.4 (*ERBB3)* | c.235T>C | p.Trp79Arg | rs891498670 |
| *2765-I-1* | *NM_000534.5 (PMS2)* | c.745_748del | p.Phe249Leufs*13 | |
|  | *NM_000124.4 (ERCC6)* | c.1670G>A | p.Arg557His | rs41549213 |
| 2072-I-1 | *NM_000400.4 (ERCC2)* | c.601C>T | p.His201Tyr | rs1799792 |
| 2036-I-1 | *NM_000400.4 (ERCC2)* | c.601C>T | p.His201Tyr | rs1799792 |
| 8040-II-1 | *NM_002769.5 (PRSS1)* | c.86A>T | p.Asn29Ile | rs111033566 |

**Supplementary Appendix 1 –** Surveillance protocol for carriers of pathogenic variants in *TP53* (including *TP53* p.Arg181Cys) based on Villani A et al. and Frebourg T et al. which is currently recommended in Israel [3,6].

1. Full physical examination – Every 6 months from birth.
2. Dermoscopy skin examination -Every year from birth.
3. Neurological examination -Every year from birth.
4. Complete blood test, tumor markers and urine hormone secretion – Every 6 months from birth.
5. Complete body (without gadolinium) and Brain (with gadolinium) magnetic resonance imaging (MRI) – Every year from birth.
6. For females, breast MRI (with gadolinium) and breast ultrasound – Every year from age of 25 or 5 years before the earliest age of diagnosis in the family (whichever is first).
7. Abdominal and pelvis ultrasound- Every 6 months from birth.
8. Colonoscopy –Every year from age of 25 or 5 years before the earliest age of diagnosis in the family (whichever is first).
